# The surgical management trends of osteoporotic vertebral compression fractures: 5-year experience in one institution

**DOI:** 10.1038/s41598-022-23106-y

**Published:** 2022-10-27

**Authors:** Jie Qin, Weiyang Zhong, Zhengxue Quan

**Affiliations:** 1 Department of Orthopaedic Surgery, The First Affiliated Hospital of Chongqing Medical University, Chongqing, China; 2 Orthopaedic Laboratory of Chongqing Medical University, Chongqing, China; 3 Department of Spine Trauma Surgery, The People Hospital of Changshou District, Chongqing, China

**Keywords:** Orthopaedics, Trauma

## Abstract

Osteoporotic vertebral compression fractures (OVCFs) have gradually become a health threat to elderly individuals. Treatment options are controversial, and many challenges remain. Our study aimed to investigate the management trends of OVCFs at a single institution, covering all cases of OVCFs between January 1, 2016, and December 31, 2020. A total of 938 OVCF patients were reviewed, and OVCFs were most common in patients over 70 years old. The hospital stay, surgery haemorrhage rate and total cost decreased year by year. The number of patients with previous OVCFs varied from 123 in 2016 to 83 in 2020. The average bone mineral density (BMD) of the patients generally decreased year by year. In OVCF treatments, the rate of PV or PK increased from 93.86% in 2016 to 98.98% in 2020, while the rate of PV combined with pedicle fixation decreased from 6.14% in 2012 to 1.12% in 2020. Most patients were treated with bisphosphonates, and only 2 patients were treated with teriparatide. The visual analogue scale scores significantly improved at the final follow-up compared with the preoperative values. The rate of previous fractures was correlated with BMD, while there were no correlations with sex, age, or anti-osteoporosis treatment. In conclusion, the 5-year incidence of OVCFs increased and average patient BMD worsened by year. Although the total cost is continuously decreasing, poor adherence to anti-osteoporosis treatments and the prevention of refracture create more severe challenges.

## Introduction

Osteoporotic vertebral compression fractures (OVCFs) occur frequently with minor trauma or even without any noticeable trauma in elderly patients, suffering from decreasing bone mineral density (BMD) or poor bone quality. OVCFs decrease quality of life because of persistent chronic back pain, worsening sagittal imbalance or even neurological impairments. The prevalence of OVCFs increases gradually with the ageing population^1–3^. Conservative treatments or cement augmentation are not perfect, and various studies have described the results, conclusions and complications of each treatment. Personalized decision-making is still needed for the optimal treatment of OVCFs^4–9^. Our study is a retrospective analysis that reported OVCF patients treated over 5 years, and it aimed to investigate the management trends in one institution.

## Materials and methods

### Patient population

This study was approved by the Institutional Review Board of the First Affiliated Hospital of Chongqing Medical University and was conducted according to the principles listed in the Declaration of Helsinki. All patients provided written informed consent. The patients between January 2016 and December 2020 were reviewed. The inclusion criteria were as follows: OVCFs diagnosed by MRI, osteoporosis diagnosed by bone mineral density (BMD) < 80 mg/cm^3^ and T score < − 2.5, and minor trauma. The exclusion criteria were as follows: patients who underwent conservative treatment, metastatic fractures, primary tumours, high-energy trauma fractures, severe compression fractures or severe kyphosis (Cobb > 30).

### Surgical techniques

According to the guidelines of the DGOU, the OVCFs were classified as OF 1–5. OF 1–2 were treated with PV, and OF 3 was treated with PK. A hybrid construct with PV/PVP and screw surgical management was recommended for OF 4^24–26^. The operations were performed by three senior surgeons. PV or PK surgery was performed bilaterally under C-arm X-ray guidance (Siemens, Germany) using sufficient local anaesthesia, and PV combined with pedicle fixation was performed under general anaesthesia. The working cannulas were successfully transpedicularly into the vertebral body. Afterwards, polymethylmethacrylate (PMMA) was slowly injected into the fractured vertebral body. The patients underwent PV combined with pedicle fixation. After cement augmentation was finished on the fractured vertebral body, the pedicle screws were fixed and locked. After surgery, negative pressure drainage was implemented, and the incision was closed. The amount of surgical haemorrhage, the surgical time, the cement volume and complications were recorded accordingly.

### Outcome assessment

For all cases, the following data were observed: the surgery time, amount of surgical haemorrhage, hospital stay, hospital cost, BMD, amount of bone cement, previous fractures and number of new fractures, visual analogue scale (VAS), etc.

### Statistical analysis

The results are expressed as the group means ± SDs. The correlation analysis was performed using the Statistical Analysis System (SAS Institute Inc., Cary, NC, USA). Differences with a *P* value < 0.05 were considered significant.

## Results

**Figure 1:**
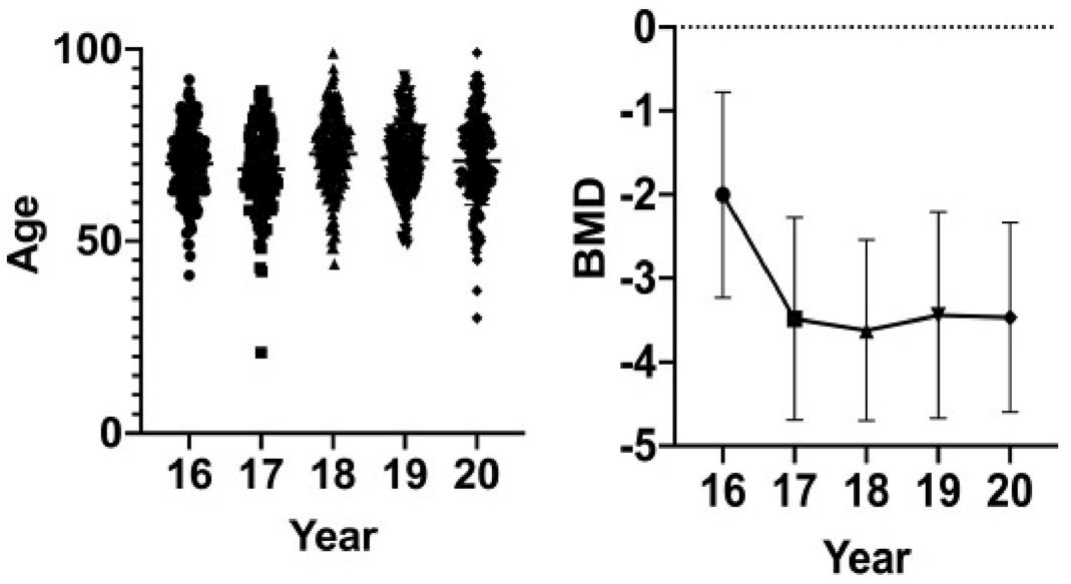
The distribution of age and BMD.

**Figure 2:**
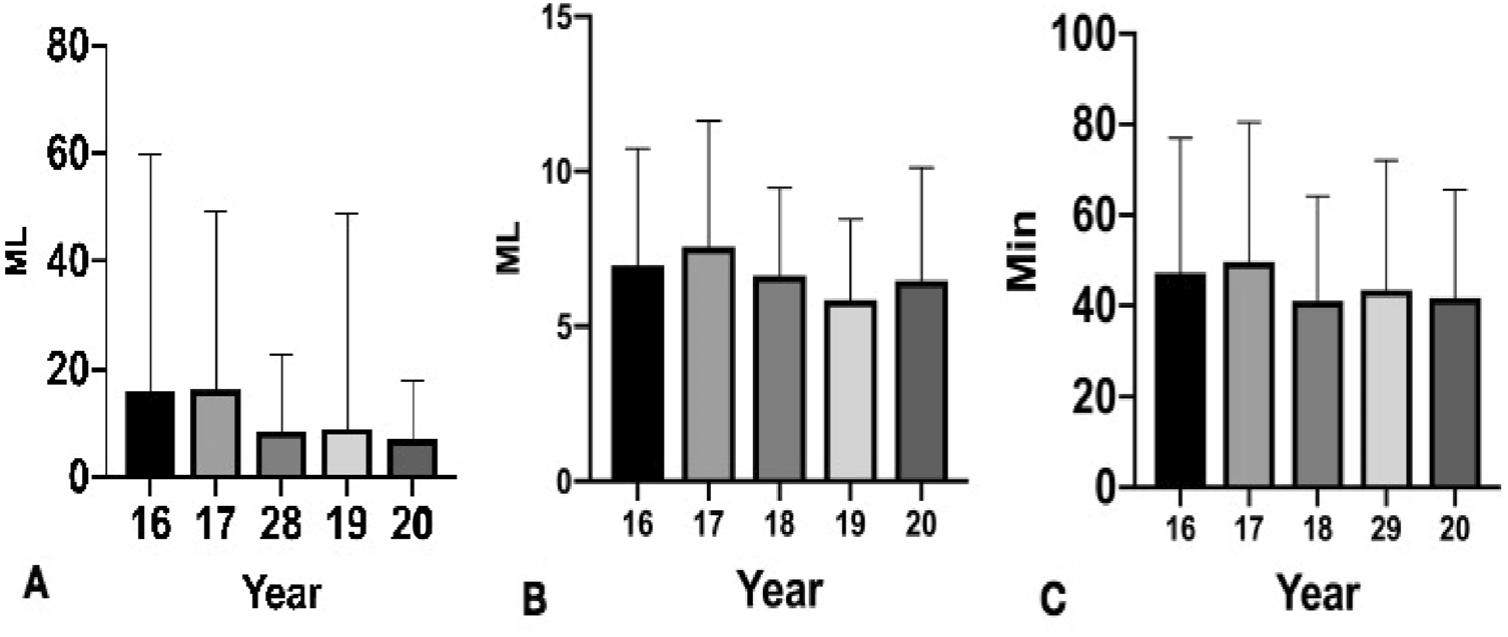
(**A**) Surgery bleeding, (**B**) cement volume and (**C**) surgery time.

**Figure 3:**
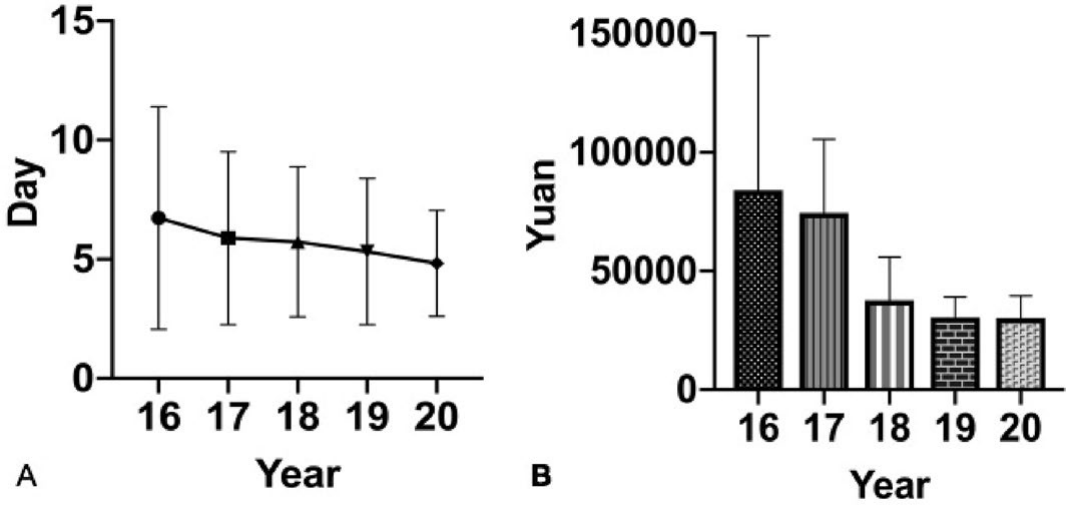
(**A**) Hospital stay and (**B**) hospital cost.

**Figure 4:**
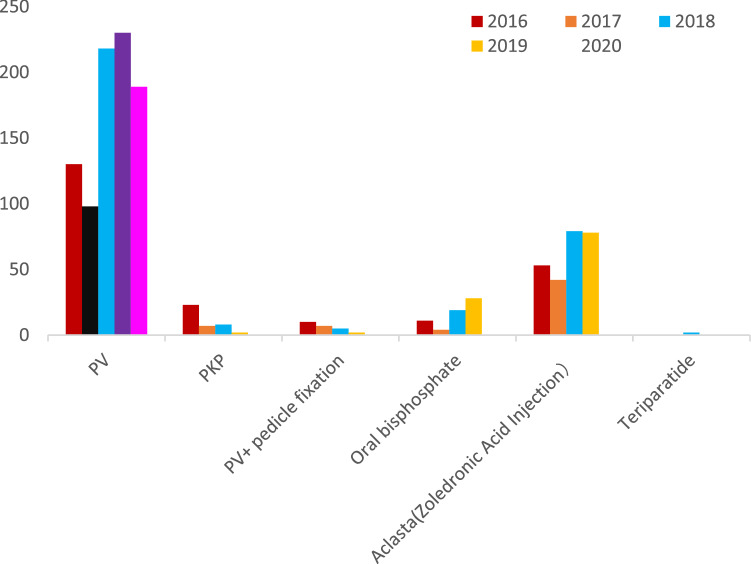
Management of OVCF patients over 5 years.

**Figure 5:**
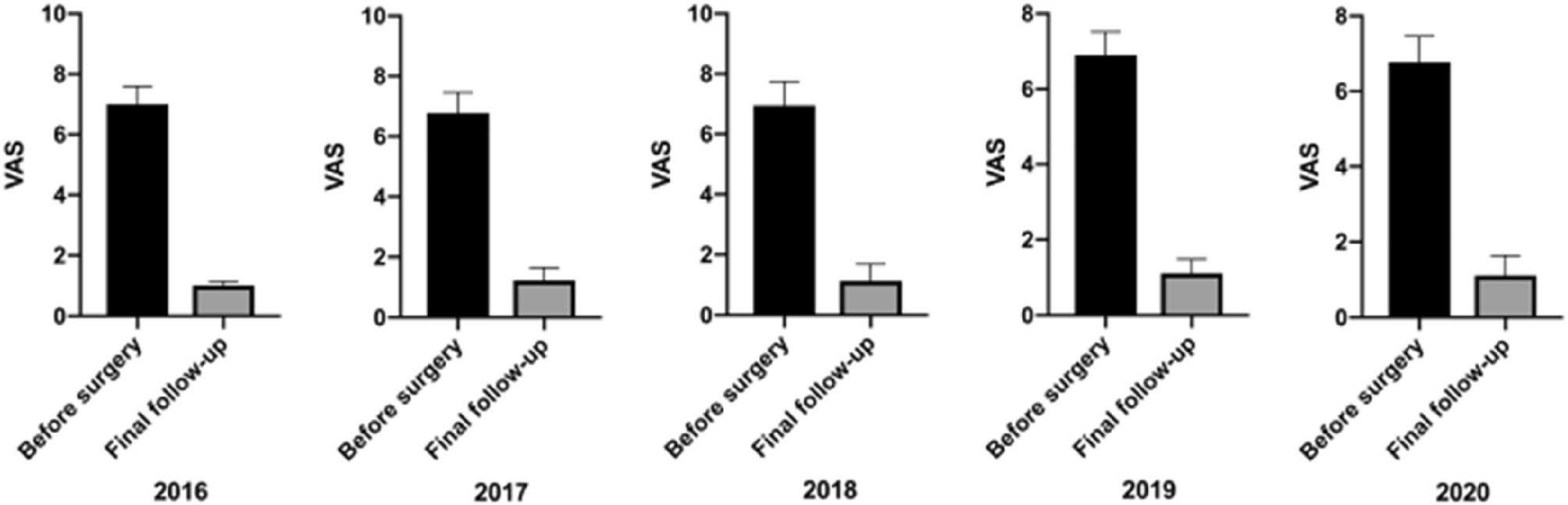
The VAS scores of OVCF patients over 5 years.

There were 938 OVCF patients from 2016 to 2020. During 2016–2020, the mean age of OVCF patients was more than 70 years except in 2017, and the incidence rate in female patients was high. OVCFs were most frequent in patients aged over 70 years (Table 1, Fig. 1). The hospital stay, surgery haemorrhage and total cost decreased year by year (Table 1, Figs. 2, 3). The number of patients with previous OVCFs varied from 123 in 2016 to 83 in 2020. The average patient BMD generally decreased year by year (Fig. 1). The fractured vertebral levels of OVCFs were mainly thoracolumbar fractures (T10-L2) (Table 2). In OVCF treatment, the rate of PV or PK increased from 93.86% (130 patients) in 2016 to 98.98% (189 patients) in 2020, while the rate of PV combined with pedicle fixation decreased from 6.14% (10 patients) in 2012 to 1.12% (2 patients) in 2020. The number of patients treated with anti-osteoporosis agents varied from 64 in 2016 to 89 in 2020. Most patients were treated with bisphosphonates, and only 2 patients were treated with teriparatide (Table 3, Fig. 4). The VAS scores significantly improved at the final follow-up compared with the preoperative values (Table 4, Fig. 5).

**Table 1:** Basic information of OVCF patients over 5 years.

	2016	2017	2018	2019	2020
Number of patients	163/1295	112/1463	231/1404	234/1619	198/1503
Male/female	40/123	30/82	52/179	47/187	35/163
Mean age (years)	70.09 ± 9.33	68.71 ± 11.26	72.70 ± 9.88	71.55 ± 9.17	70.87 ± 11.41
Hospital stay (days)	6.73 ± 4.66	5.89 ± 3.63	5.73 ± 3.15	5.32 ± 3.08	4.83 ± 2.22
Surgery time (min)	47.25 ± 29.73	49.66 ± 30.78	41.03 ± 23.10	43.47 ± 28.54	41.67 ± 23.84
Amount of bone cement (ml)	6.96 ± 3.76	7.56 ± 4.08	6.63 ± 2.84	5.85 ± 2.61	6.47 ± 3.65
Surgery haemorrhage (ml)	15.94 ± 43.78	16.78 ± 33.10	8.50 ± 14.20	8.98 ± 39.63	7.07 ± 10.67
Hospital cost (yuan)	83,945 ± 65,008	74,430 ± 30,957	37,726 ± 18,027	30,407 ± 8687	30,157 ± 9405
Previous OVCFs	123/163	43/112	102/231	122/234	83/198
BMD	− 2.00 ± 1.23	− 3.48 ± 1.21	− 3.62 ± 1.08	− 3.44 ± 1.23	− 3.47 ± 1.13

**Table 2:** Fractured vertebral levels in OVCF patients over 5 years.

	2016	2017	2018	2019	2020
T1-T9	15	15	23	16	42
T10-L2	116	90	183	203	145
L3-L5	32	5	25	15	11

**Table 3:** Surgical and drug management of OVCF patients over 5 years.

	2016	2017	2018	2019	2020
PV	130	98	218	230	189
PKP	23	7	8	2	7
PV + pedicle fixation	10	7	5	2	2
Oral bisphosphate	11	4	19	28	27
Aclasta (Zoledronic Acid Injection)	53	42	79	78	62
Teriparatide	0	0	2	0	0

**Table 4:** VAS score of OVCF patients over 5 years.

	2016	2017	2018	2019	2020
**VAS**					
Before surgery	7.00 ± 0.59	6.78 ± 0.68	6.95 ± 0.72	6.91 ± 0.61	6.88 ± 0.67
Final follow-up	1.02 ± 0.13*	1.23 ± .0.42*	1.13 ± 0.56*	1.11 ± 0.37*	1.12 ± 0.52*

*Before treatment vs. final follow-up, *P* < 0.05.

The rate of previous fractures was correlated with BMD (r = 0.9431, *P* = 0.0161) and was not correlated with sex (*P* = 0.9452), age (*P* = 0.9313), or anti-osteoporosis treatment (*P* = 0.3503).

## Discussion

With the ageing of society, the incidence rate of osteoporosis is increasing year by year. Low energy trauma or daily life can lead to OVCFs, causing pain, kyphosis, activity limitation and even death. Elderly individuals face a more severe decrease in quality of life. The results of the seventh national census showed that the population aged 60 and above is 264.02 million (18.70%), of which the population aged 65 and above is 190.64 million (13.50%)^10–12^. In our study, the mean age of the patients was more than 70 years except in 2017, and was greater than 65 years in all years.

At present, the elderly mostly live alone and have insufficient care at home, which affects the incidence of OVCFs. Although there is an increasing number of care institutions, they still need professional medical care. There are also some elderly individuals who help their children raise their grandchildren, which especially requires more physical labour and faces a greater chance of minor energy trauma^13–15^. They also face more economic pressure, which is one of the reasons for their poor compliance with anti-osteoporosis drugs^16–18^. Few patients comply with teriparatide treatment, which is very expensive for elderly retired at the age of 60. The retirement salary is still very low, so it is still very difficult to manage higher medical expenses. Many patients are farmers who do not have retirement wages and rely entirely on the economic income of their children for support.

From 2016 to 2020, with the improvement of surgical technology and perioperative management, the hospital stay, surgical bleeding and hospitalization expenses decreased year by year, which could decrease the economic burden on family and health authorities. More patients underwent minimally invasive treatments, such as PV or PK, which result in rapid postoperative recovery. If vertebral compression is serious and the stability of the spine is damaged, internal fixation is suggested^19–26^.

From 2016 to 2020, average patient BMD worsened over the years, which aroused our concern, although inpatient treatments have greatly improved. It was speculated that it may be that many elderly people did not pay attention to the treatment of anti-osteoporosis and did not receive early detection and often had BMD tests after OVCFs. In addition, because of the great economic pressure on elderly individuals, they had very poor compliance with the treatment of anti-osteoporosis, which was what we learned and confirmed among hospitalized patients. As a city in western China, the low per capita income and per capita medical expenses of Chongqing were also an important reason for low compliance.

Our study had several limitations. First, OVCF patients who underwent conservative treatment were excluded. Second, there was no age-stratified analysis. Third, the study of a single centre is still limited, and more centres and sample sizes will be included in future studies.

In conclusion, the 5-year incidence of OVCFs increased, and BMD worsened year by year. Although the total cost is continuously decreasing, poor adherence to anti-osteoporosis treatments and the prevention of refracture create more severe challenges.

## Data Availability

The datasets used and/or analysed during the current study are available from the corresponding author on reasonable request.
